# Supplementary material for: Deep Learning Supplants Visual Analysis by Experienced Operators for the Diagnosis of Cardiac Amyloidosis by Cine-CMR
Source: Diagnostics (Basel). 2021 Dec 29;12(1):69. doi: 10.3390/diagnostics12010069 (PMC8774777; doi:10.3390/diagnostics12010069)
Supplement: Supplementary file 1 [file diagnostics-12-00069-s001.zip › diagnostics-1525234-supplementary.pdf]

Table supp1: Clinical and CMR characteristics of AL and TTR cardiac amyloidosis

	**AL**	**TTR**	**P**
N	59	38	
Age (years)	72.39±9.09	74.12±9.26	0.40
Sex (F/M)	19/40	4/34	0.005
Weight (kg)	68.60±15.47	76.29±14.80	0.024
Height (m)	170.14± 9.04	172.11±8.37	0.31
BSA (m^2)	1.82±0.21	1.91±0.18	0.04
IVS (mm)	16.74±3.07	20.00±3.49	0.0001
LVMI (g/m^2)	106.54±29.64	130.31±27.55	0.0003
LVDVI (ml/m^2)	66.47±22.86	74.03±21.65	0.12
LVEF (%)	59.85±9.54	58.20±11.26	0.46
LA surface (cm^2)	30.22±4.49	30.57±3.54	0.77
Systolic time (ms)	312±44	333±35	0.076
T1 (ms)	1142.9±52.9	1141±45.3	0.90
ECV (%)	49.53±8.51	58.60±10.94	<0.017
N frames long axis/patient	2.22±1.12	2.28±0.78	0.78
N frames short axis/patient	3.38±1.55	3.56±1.44	0.59
N frames/patient	5.60±2.04	5.83±1.76	0.58
N frame post-gadolinium	94/330	52/222	ns
Pericardial effusion	36 (61%)	7 (18%)	0.0004
Pleural effusion	28 (47.5%)	6 (16%)	0.014
Both effusions	16 (27%)	2 (5%)	0,007

Characteristics of patients with AL and TTR amyloidosis (same abbreviations as in table 1).
